# Bridging the Gap Between Vessels and Nerves in Fabry Disease

**DOI:** 10.3389/fnins.2020.00448

**Published:** 2020-06-16

**Authors:** Julia Forstenpointner, Manon Sendel, Paul Moeller, Maren Reimer, Sima Canaan-Kühl, Jens Gaedeke, Stefanie Rehm, Philipp Hüllemann, Janne Gierthmühlen, Ralf Baron

**Affiliations:** ^1^Division of Neurological Pain Research and Therapy, Department of Neurology, University Hospital Schleswig-Holstein, Campus Kiel, Kiel, Germany; ^2^Department of Medicine, Division of Nephrology, Charité, Berlin, Germany

**Keywords:** lysosomal storage disease, laser speckle contrast analysis, LASCA, Quantitative Sensory Testing, QST

## Abstract

**Purpose:**

Fabry disease frequently includes pain as an early disease feature, which was characterized as a dysfunctional processing of somatosensory information in various studies. The pathomechanism involves the mutation in the x-chromosomal GLA-gene and a consequent reduction of the α-galactosidase. This results in an insufficient reduction of globotriaosylceramide (GL3). Interestingly, an accumulation of GL3 was shown in both vascular endothelial cells and nerve tissue. This implicates that both an endothelial and nerve-dependent dysfunction may be considered as prominent mechanisms in pain pathogeneses.

**Patients and Methods:**

The exploration of endothelial and C-fiber-dependent microcirculatory changes was conducted in a healthy cohort (*n* = 22) and in patients with polyneuropathy (*n* = 21) and Fabry disease (*n* = 15). Microcirculatory measurements were conducted using a laser speckle contrast analysis (LASCA) in combination with a thermoprobe controlling system, which applied a constant heat stimulus (42°C). Additionally, nerve fiber function was assessed via Quantitative Sensory Testing and heart rate variability (HRV).

**Results:**

The results indicated a characteristic perfusion profile in the control group as well as both patient groups. Fabry patients had the smallest increase of endothelial-dependent perfusion as compared to the others [% increase as compared to Fabry: control + 129% (*p* = 0.002), PNP + 126% (*p* = 0.019)]. The sensory testing indicated a dysfunctional processing of A-delta fibers in Fabry disease as compared to healthy controls [cold detection threshold (CDT): *p* = 0.004, mechanical pain threshold (MPT): *p* = 0.007] and PNP patients (MPT: *p* = 0.001).

**Conclusion:**

Our results point to both an endothelial and a nerve-dependent dysfunction in Fabry disease. Therefore, not only direct changes in nerve fiber tissue may contribute to an altered sensory processing. Indeed, evidence of a perfusion change in vasa nervorum could also contribute to the dysfunctional processing of sensory information, which likely occurs under physical stress.

## Introduction

In the past, various studies have aimed to unravel the pain pathogeneses in Fabry disease. The underlying pathomechanism involves a mutation in the x-chromosomal galactosidase-α-gene(GLA-gene), which encodes for α-galactosidase(α-GAL) (Calhoun et al., 1985; Gal, 2010). The decrease of functioning α-GAL consequently leads to an accumulation of globotriaosylceramide (GL3) in different organs and tissues such as the heart, the kidney, blood vessels, and nerve fibers (Calhoun et al., 1985; Kaye et al., 1988; El-Abassi et al., 2014). Although the molecular pathogenesis of the disease is well established, the pathophysiology of pain development remains unclear yet. Interestingly, with regard to disease development, studies indicated an involvement of both nerve fiber and endothelial dysfunction. Contributing to a nerve-dependent mechanism, the characterization of dysfunctional somatosensory processing in Fabry disease revealed four different pain modalities that included (i) evoked pain (43%), (ii) spontaneous pain (33%), (iii) neuropathic pain (12%), and (iv) temperature-dependent pain (12%) (Uceyler et al., 2014). Moreover, a dysfunction of small fibers was shown via sensory testing, which, however, also revealed the selective integrity of A-beta fiber-dependent parameters (Maag et al., 2008). In line with this, pain-related evoked potentials (i.e., PREPs), which assumable assess A-delta fiber integrity, indicated a decrease of amplitudes in Fabry patients, which was further pronounced with increasing disease severity (Uceyler et al., 2013). Moreover, a study assessing A-delta laser evoked potentials (i.e., LEPs) in Fabry patients confirmed a decrease in amplitudes as compared to healthy controls (Valeriani et al., 2004). Other studies evaluating nerve fiber histopathology in Fabry disease found degenerative morphological changes in nerve fiber tissues as well as lipid inclusions (Scott et al., 1999). Additionally, an accumulation of glycosphingolipids in spinal and sympathetic ganglia (Hozumi et al., 1989) as well as an enlargement of dorsal root ganglia and a decrease of blood perfusion (Gadoth and Sandbank, 1983; Godel et al., 2017, 2018, 2019) was found in Fabry disease. However, if the latter findings represent a pathognomic feature of Fabry or if the morphometric changes rather represent an unspecific marker for storage diseases, remains unclear yet. Contributing to this assumption, an enlargement of sacral dorsal root ganglion was demonstrated in hereditary transthyretin (ATTRm) amyloidosis (Masuda et al., 2018). However, either way, the ganglionic as well as peripheral nerve fiber tissue modifications may potentially contribute to pain development.

Additionally, to alterations of nerve fiber tissue, an endothelial modification of blood vessels was shown in Fabry disease. Several studies indicated a significant increase of intima-media thickness of carotid as well as radial arteries (Boutouyrie et al., 2001; Barbey et al., 2006; Kalliokoski et al., 2006). These findings were confirmed histopathological by proving a glycosphingolipid accumulation in blood vessel walls (Ferreira and Gahl, 2017). Moreover, an altered endothelial nitric oxide (NO) pathway was shown in an alpha-galactosidase A-knock out mouse model of Fabry disease and in a study investigating cerebral and dermal blood vessels in Fabry patients (Moore et al., 2001; Shu et al., 2009). However, whether the glycolipid-associated vasculopathy may enhance nerve fiber tissue damage via a dysfunctional perfusion of vasa nervorum or if the accumulation of lipids in nerve fiber tissue is the prevalent mechanism for pain generation remains unsolved yet. In fact, also a combination of both mechanisms seems possible in Fabry disease.

The aims of the study were as follows: (i) to investigate both C-fiber and endothelial dependent microcirculation changes in Fabry patients via functional laser speckle contrast analysis (fLASCA), (ii) to characterize the sensory profile as well as autonomic nervous function in Fabry disease, and (iii) to detect sensory/endothelial parameters indicative for Fabry disease as compared to PNP and healthy controls.

## Materials and Methods

### Study Design

This explorative pilot study included 15 patients with Fabry disease [m/f: 5/10; age (±SD): 45.2 (± 18.1)], 21 patients with polyneuropathy [m/f: 10/11; age (±SD): 64.4 (±11.5)], and 22 healthy volunteers [m/f: 9/12; age (±SD): 45.9 (±19.5)].

All patients received the same testing protocol, which included a Quantitative Sensory Testing (QST), a heart variability frequency measurement, and a fLASCA assessment. All QST and fLASCA assessments were performed at the dorsomedial foot within the area of neuropathy in patients or the respective control area in healthy volunteers. All assessments were performed within a single visit at the same study site, i.e., the Department of Neurology at University Hospital of Schleswig-Holstein, Campus Kiel. The general exclusion criteria encompass the exclusion of all participants with current alcohol or drug abuse as well as pregnant or lactating women. Healthy subjects were only eligible for participation, when not suffering from any neurological disorders, pain disorders, severe conditions of the psyche, or any other severe organ system failure. Additionally, healthy subjects were excluded if suffering from any severe pain or due to intake of any analgesic medication within the past 14 days. The inclusion criteria for polyneuropathy diagnosis were based on the consensus criteria for a symmetric polyneuropathy (ordinal likelihood + + + +) (England et al., 2005). The inclusion criteria for Fabry diagnosis required a mutation in the GLA-gene as well as the clinical evaluation via two independent sites specialized in Fabry disease (i.e., Kiel and Berlin). Patient characteristics are displayed in Table 1.

**TABLE 1:** Characterization of patients.

Number	Polyneuropathy		Fabry		
		
	Age/gender	Etiology	Age/gender	cDNA Change	Location
1	75/f	PNP (diabetic)	56/f	Del 15bp codon 354–359^P^	Exon 7
2	56/f	PNP (diabetic)	29/f	c.937G > T^U^	Exon 6
3	41/f	PNP (HMNS II)	58/f	Unknown	Intron
4	54/f	PNP (unknown)	52/f	Unknown	Intron
5	61/m	PNP (paraneoplastic)	56/f	c.937G > T^U^	Exon 6
6	53/m	PNP (CIDP)	49/m	c.607G > A^P^	Exon 4
7	68/m	PNP (paraneoplastic)	70/m	c.937G > T^U^	Exon 6
8	60/m	PNP (diabetic)	55/f	c.937G > T^U^	Exon 6
9	79/f	PNP (unknown)	53/f	c.902G > A^P^	Exon 6
10	65/f	PNP (unknown)	14/m	c.902G > A^P^	Exon 6
11	77/f	PNP (diabetic)	14/m	c.902G > A^P^	Exon 6
12	79/f	PNP (diabetic)	17/f	c.902G > A^P^	Exon 6
13	77/m	PNP (unknown)	57/f	c.101A > G^P^	Exon 1
14	65/m	PNP (unknown)	59/f	c.416A > G^LB^	Exon 3
15	80/m	PNP (post alcohol misuse)	39/m	c.937G > T^U^	Exon 6
16	59/f	PNP (post chemotherapy)			
17	67/m	PNP (post chemotherapy)			
18	62/m	PNP (unknown)			
19	54/f	PNP (unknown)			
20	45/f	PNP (unknown)			
21	75/m	PNP (unknown)			

*The characterization of polyneuropathy and Fabry patients indicates information on age, gender, and etiology/genetics. PNP: polyneuropathy, CIDP: chronic inflammatory demyelinating polyneuropathy, HMNS II: hereditary motor and sensory neuropathy II, f: female, m: male, ^*P*^: pathogenic, ^*U*^: uncertain, ^*LB*^: likely benign (variant classification was obtained from https://www.centogene.com/centolsd.html).*

Prior to the study, the aim and nature of the tests were explained to the subjects in accordance with the Declaration of Helsinki and all patients gave written informed consent.

The study was approved by the Ethics Committee of the University Hospital of Kiel (study protocol number: A 101/15). The STROBE guidelines were applied for data presentation (von Elm et al., 2007).

### Quantitative Sensory Testing

The QST assesses integrity of small and large fiber function according to the protocol of the German Research Network on Neuropathic Pain (DFNS) (Rolke et al., 2006; Magerl et al., 2010; Pfau et al., 2012). The shortened protocol included a thermal sensory testing conducted by a thermotest device (TSA 2001-II, Medoc, Israel), which applied heat and cold stimuli (ramp 1°C/s; max 50°C, min 0°C). Thereby, thermal detection [i.e., warm detection threshold (WDT) and cold detection threshold (CDT)] as well as pain thresholds [i.e., heat pain threshold (HPT) and cold pain threshold (CPT)] evaluated C- and A-delta fiber integrity. Further on, the mechanical sensory testing composed a pinprick testing [i.e., mechanical pain threshold (MPT)] assessing A-delta fiber function. Moreover, A-beta fiber integrity was evaluated [i.e., mechanical detection threshold (MDT)] by applying different forces of light touch (Fruhstorfer von Frey hair; Optihair2-Set, Marstock Nervtest, Germany) and the assessment of the vibration detection threshold (VDT) by a standard neurological tuning fork (64 Hz). The interpretation of nerve fiber integrity, i.e., gain or loss of function, was conducted via the transformation of parameters into *z* values, according to a previously published algorithm (Magerl et al., 2010). The parameters exceeding the *z* value of +1.96 indicate a gain of nerve fiber function, whereas parameters below −1.96 indicate a loss of function. Thereby, this algorithm allows to control for age-, gender-, and localization-specific sensory changes.

### Heart Rate Variability (HRV)

The heart rate variability (HRV) of subjects was recorded via a three-lead electrocardiogram. Parameters of HRV were assessed while resting for a period of 5 min [VC_r (Coefficient of variation), RMSSD_r (root mean square of successive square differences)] and during controlled breathing for a period of 110 interbeat intervals [VC_b (Coefficient of variation_breath), RMSSD_b (root mean square of successive square differences_breath), MCR (Mean Circular Resultant_breath)]. A computer-assisted equipment and software (ProSciCard III, MediSyst GmbH, Germany), developed according to the 1996 Task Force Guidelines, was used to process and analyze Heart Rate Variability [HRV] (1996). This analysis algorithm detected artifacts and extrasystoles as well as dismissed a series with an artifact percentage of >10% (Ziegler et al., 1992).

### Functional Laser Speckle Contrast Analysis

In order to assess functionality of both C-fiber and endothelial-dependent microcirculation changes of the skin, the method of fLASCA was applied. The novelty of this method comprises the combination of previously established principles and methods that aimed to investigate C-fiber and endothelial integrity. The reliability and the physiology of these methods [i.e., single-probe laser Doppler perfusion measurements or laser speckle contrast analysis (LASCA)] were assessed in a series of animal and human studies (Kellogg, 2006; Widmer et al., 2007; Johnson and Kellogg, 2010; Nieuwenhoff et al., 2016; Kubasch et al., 2017). Further on, the principle of fLASCA involves a continuous heating of the skin (until 42°C), which initially leads to a release of calcitonin gene-related peptide (=CGRP) from C-fiber axon endings. Second, there is an endothelial-dependent increase of blood perfusion mediated via activation of eNOS (endothelial NO synthase 3) (Kellogg, 2006). The skin was heated by a circular thermoprobe (probe 415-339, Perimed, Stockholm, Sweden), which was filled with water and regulated by a temperature controlling system (PF 5020, Perimed, Stockholm, Sweden). The measurement was conducted within a timeframe of 40 min, consisting of (i) a 5-min adaption measurement (individual skin temperature), (ii) a 10-min baseline measurement (32°C skin temperature) followed by (iii) a 25-min-long period of constant skin heating (42°C skin temperature). In comparison to previous studies, evaluating heat-dependent microcirculation changes, the underlying method was able to evaluate the C-fiber/endothelial microcirculation of an entire area instead of a punctual single probe perfusion measurement.

### Statistical Analysis

All parameters were displayed as mean (±SEM). In order to calculate differences between patient/healthy volunteer groups, the statistical analysis of fLASCA, QST, and HVF parameters was conducted via Mann–Whitney *U*-test. In-between subject comparisons within the same group were calculated via Wilcoxon signed rank test (SPSS 23.0; SPSS, Inc., Chicago, IL, United States). Non-parametric tests were applied due to non-normal distribution (i.e., Shapiro–Wilk test) and relatively small sample size. *p*-values < 0.05 were considered as statistically significant.

## Results

### Sensory Profiles Indicate A-Delta Fiber Impairment in Fabry Disease

**FIGURE 1:**
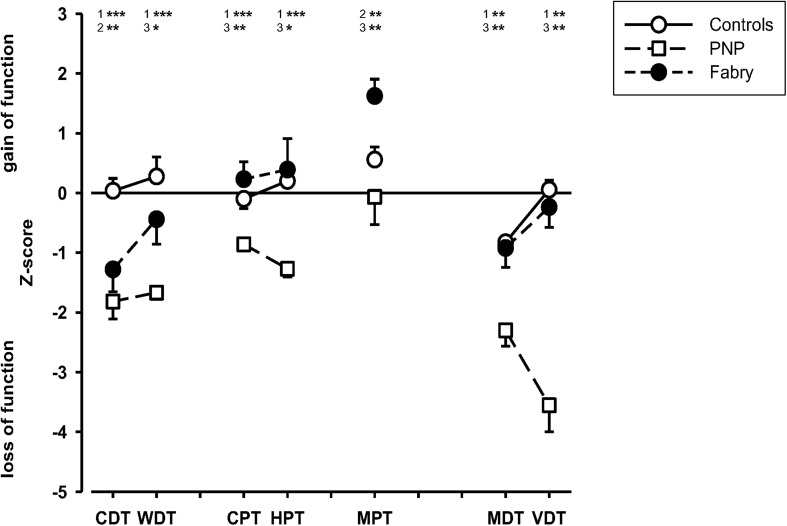
Sensory profiles of patients and healthy volunteers. Displayed are the sensory profiles of the control group, the polyneuropathy patients, and the Fabry patients. The sensory profile indicates an A-delta fiber dysfunction but an A-beta and C-fiber integrity in Fabry patients, whereas in PNP patients, results indicate a generalized loss of function in small and large fiber afferents. CDT (cold detection threshold), WDT (warm detection threshold), CPT (cold pain threshold), HPT (heat pain threshold), MPT (mechanical pain threshold), MDT (mechanical detection threshold), VDT (vibration detection threshold). 1 = control vs. PNP; 2 = control vs. Fabry; 3 = PNP vs. Fabry. Mann–Whitney *U*-test; *p < 0.05; **p < 0.01; ***p < 0.001.

The sensory testing indicated integrity of parameters (i.e., MDT and VDT) assessing A-beta fiber function in Fabry disease as compared to the sensory profile of polyneuropathy patients. In contrast, patients with Fabry disease showed a dysfunctional processing in parameters assessing A-delta fiber function as compared to the controls, indicating hyposensitivity in cold detection (CDT, *p* = 0.004) and hypersensitivity toward mechanical pain stimuli (MPT, *p* = 0.007). When comparing these parameters in Fabry and PNP patients, CDT did not differ significantly whereas MPT indicated a significant hypersensitivity toward pinprick stimuli in Fabry (MPT, *p* = 0.001). Parameters assessing C-fiber integrity (i.e., WDT, HPT, and CPT) revealed no difference in Fabry patients in comparison to healthy controls, but indicated significantly less hyposensitivity as compared to polyneuropathy patients (WDT, *p* = 0.039; HPT, *p* = 0.012; CPT, *p* = 0.003) (Figure 1).

### HRV Suggests Integrity of Autonomic Innervation in Fabry Disease

**FIGURE 2:**
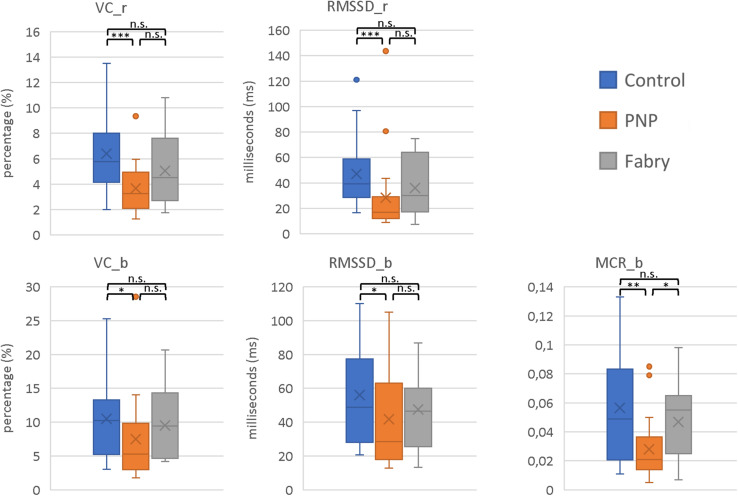
Parameters of HRVs. Displayed are the HRV parameters VC_r, RMSSD_r, VC_b, RMSSD_b, and MCR_b. In all parameters, the most pronounced decrease in HVF was shown in polyneuropathy patients. Moreover, these results indicate integrity of autonomic C-fiber pathways in Fabry disease. VC_r (Coefficient of variation_rest); RMSSD_r (root mean square of successive square differences_rest); VC_b (Coefficient of variation_breath); RMSSD_b (root mean square of successive square differences_breath); MCR (Mean Circular Resultant_breath). Mann–Whitney *U*-test; ^∗^*p* < 0.05; ^∗∗^*p* < 0.01; ^∗∗∗^*p* < 0.001; n.s., not significant.

The parameters VC_r (Coefficient of variation_rest) (*p* < 0.001), RMSSD_r (root mean square of successive square differences_rest) (*p* < 0.001), VC_b (Coefficient of variation_breath) (*p* = 0.027), RMSSD_b (root mean square of successive square differences_breath) (*p* = 0.049), and MCR (Mean Circular Resultant_breath) (*p* = 0.018) indicated a decreased HRV in polyneuropathy patients as compared to healthy controls. Interestingly, no significant difference was shown between controls and Fabry patients, suggesting less impairment of the autonomic nervous pathways. However, these results have to be interpreted with caution due to the age difference between Fabry (mean age, 45.2) and polyneuropathy (mean age, 64.4) patients (Figure 2).

### fLASCA Indicates a Dysfunctional Endothelial NO Release in Fabry Disease

**FIGURE 3:**
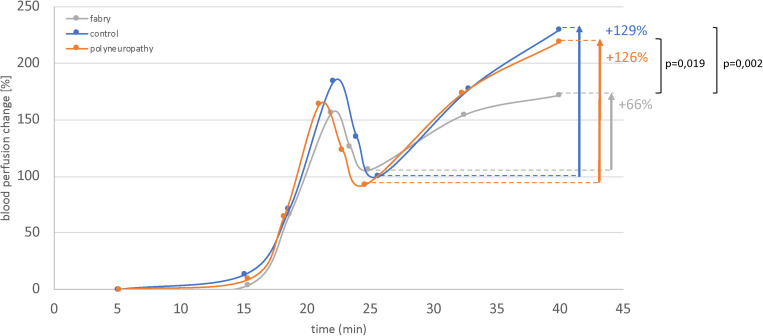
Functional laser speckle contrast analysis profiles. Displayed are the fLASCA profiles of the healthy volunteers and the polyneuropathy and Fabry patients. The eNOS-dependent blood perfusion changes (i.e., period of time: 25–45 min) were significantly decreased in Fabry patients as compared to healthy controls and PNP patients. This altered NO metabolism may also affect vasa nervorum. Mann–Whitney *U*-test.

**FIGURE 4:**
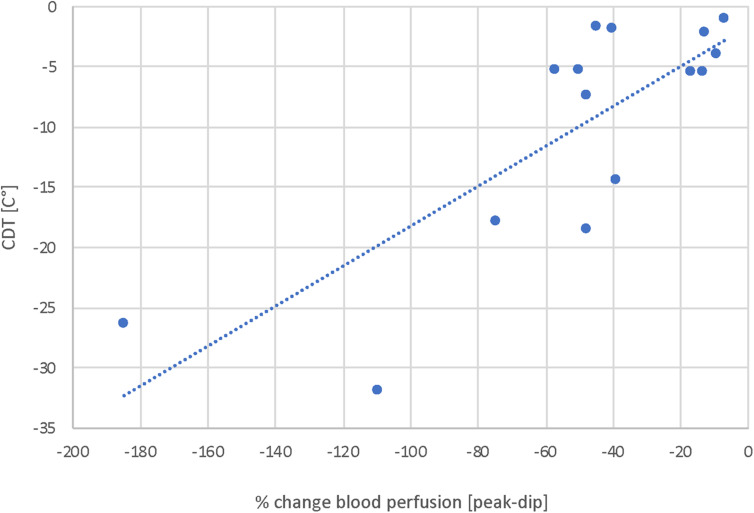
Correlation analysis of blood perfusion change (%) and CDT. Displayed is the correlation of blood perfusion change [peak-dip%] and A-delta fiber integrity. These results suggest a correlation of A-delta fiber integrity and NO-dependent vasoresponse mechanisms and thereby suggest an interrelation of peripheral nerves and vessels. Spearman Rho, *p* = 0.007, Rho = 0.663.

The results of the fLASCA indicated a characteristic change in blood perfusion in the healthy volunteer group and in both patient groups. The fLASCA profile comprised (i) a C-fiber-dependent increase in blood flow (i.e., peak), (ii) followed by a short decrease of blood perfusion (i.e., dip), and (iii) a subsequent endothelial NO-dependent vasodilatation (i.e., plateau). Interestingly, the fLASCA profiles of Fabry patients showed a decreased blood perfusion change in the plateau, indicating a dysfunctional release of endothelial NO [Fabry vs. control (*p* = 0.002), Fabry vs. PNP (*p* = 0.019), control vs. PNP (*p* = 0.474)] (Figure 3). Other than that, no significant differences between groups were detected in C-fiber-dependent blood perfusion changes. Interestingly, only in Fabry patients was the % change in blood perfusion between peak and dip (i.e., peak–dip%) significantly correlated to the cold detection threshold (C°) (Spearman Rho, *p* = 0.007, Rho = 0.663) (Figure 4).

## Discussion

Pain is a frequent symptom of Fabry disease, occurring already at early stages. So far, not only damage of nerve fiber tissue has been reported, a significant portion of studies have also indicated a dysfunctional endothelial metabolism. However, the characteristic sensory profiles in Fabry disease have not been studied with regard to the endothelial function yet. Moreover, to understand the underlying mechanisms of pain, it is important to study what distinguishes Fabry disease not only from healthy controls. It is most important to investigate which parameters distinguish Fabry from other painful conditions such as polyneuropathy. In that sense, understanding the underlying pain mechanisms could pave the way for more specific treatment strategies in painful Fabry conditions.

Three important findings of this study can be summarized:

1. Sensory profiles indicated a dysfunctional processing of A-delta fiber-mediated stimuli in Fabry disease as compared to healthy volunteers.
2. Measures of HRV suggest integrity of autonomic C-fiber pathways in Fabry disease.
3. The fLASCA indicates a dysfunctional endothelial NO release in Fabry disease.

### Integrity of C-Fiber Pathways in Fabry Disease

In general, our results suggest an integrity of C-fiber pathways in Fabry disease. This notion was confirmed by two independent testing results. First sensory testing revealed *z* values very close to normative values (i.e., close to 0) in the WDT, HPT, and CPT parameters. These results are fairly in line with a previous study investigating small fiber integrity with sensory testing (Maag et al., 2008). Second, HRV indicated that in Fabry disease, autonomic C-fibers may not underlie such alteration as in polyneuropathy. Interestingly, a similar result indicated almost normal autonomic cardiovascular function in Fabry disease, underpinning that an autonomic neuropathy seems to play a subordinate role, unlike in polyneuropathies (Biegstraaten et al., 2010). However, these results need to be interpreted with caution due to the age difference between Fabry (mean age, 45.2) and polyneuropathy (mean age, 64.4) patients.

Our results suggest that the underlying pathomechanisms in Fabry disease, specifically target A-delta fiber afferents, therefore may rather implicate a central disinhibition pain mechanism due to a reduced A-delta fiber input. Another study pointing to this exact mechanism of action investigated A-delta- and C-fiber integrity in Fabry patients via LEPs. This study found a reduced A-delta-LEP amplitude and suggested a relative overflow of C-fiber input (Valeriani et al., 2004). Both previous results and our results are therefore in line with the concept of a central disinhibition pain mechanism that assumes a reduced protective A-fiber inhibition signal but a constant physiological peripheral C-fiber input (Melzack and Casey, 1968; Craig et al., 1994). Therefore, such a mechanism may implicate a fundamentally different therapy concept, due to the fact that these pain conditions are conveyed via the medial thalamic pathway and thereby interact with basic central networks such as salience, reward, and antireward (Downar et al., 2003; Leknes et al., 2011).

### The Role of the Dysfunctional Endothelial NO Metabolism in Fabry Disease

So far, the role of endothelial vasculopathies in Fabry disease was only studied with regard to vasodilatation in medial and large arterial vessels. The uncoupling of the eNOS mechanism, however, is associated with the loss of a physiological vasodilatation. Recently, a study suggested the coupling of GLA and eNOS by investigating the knockdown of GLA expression in CRISPR/Cas9 (genome edited via Clustered Regularly Interspaced Short Palindromic Repeats) GLA-deficient cells (Kaissarian et al., 2018). However, GL3 has also been suggested to accelerate endocytosis and lysosomal degradation of K_*Ca*_3.1 via a clathrine-dependent process, fostering endothelial dysfunction (Choi et al., 2014). Still, *in vivo* human data on the endothelial NO metabolism in Fabry disease are rare. Therefore, the presented study is one of the first to investigate the blood perfusion changes, *in vivo* in the microcirculatory metabolism. Additionally, it compares the microcirculatory and sensory properties in Fabry disease with those of PNP patients and healthy controls. Our findings suggest a correlation of vascular response mechanisms and A-delta fiber integrity in Fabry disease. This correlation was not found in the PNP and the control group. However, the decrease of blood perfusion is probably related to the characteristic pathophysiological vasculopathy, altering NO liberation. Such a maladaptive vascular response could be tentatively related to pain conditions associated with physical stress in Fabry. In line with this notion, Politei et al. (2016) suggested that the application of capsaicin cream or lidocaine may reduce physical stress induced pain in Fabry patients. In particular, capsaicin leads via its CGRP-releasing mechanism to a distinct increase in blood perfusion and thereby could attenuate these specific pain conditions (Hughes and Brain, 1991; Baron et al., 1999; Geber et al., 2007).

Interestingly, studies also pointed to an altered sympathetic innervation as well as noradrenergic/cholinergic neurotransmitter coding in Fabry disease (Stemper and Hilz, 2003; Jardim et al., 2006; Kaneski et al., 2016). In this context, an *in vitro* model for neuronal dysfunction in Fabry disease suggested insufficiency of cholinergic function (Kaneski et al., 2016). It was also shown that during local and whole-body heating, acetylcholine is released, inducing cutaneous vasodilation, presumably via NO synthase pathways (Shibasaki et al., 2002; Holowatz et al., 2005). Although the current study did not explicitly address this objective, our findings in combination with previous evidence may suggest that cholinergic nerve fiber dysfunction contributes to the altered NO metabolism in Fabry disease. Moreover, a dysfunctional cholinergic response may explain the characteristic general heat intolerance observed in Fabry patients (Shelley et al., 1995; Mehta et al., 2010), i.e., due to its co-regulation in response to whole-body heating (Shibasaki et al., 2002; Johnson et al., 2014).

### Conclusion

Our results indicate a decrease in the NO-dependent microcirculatory fLASCA profile as well as a correlation of the cold perception and vascular adaption processes in Fabry patients. In fact, these effects might be interrelated and a dysfunctional perfusion of vasa nervorum may enhance an altered sensory processing. The characteristic pain condition in Fabry disease might therefore originate from a central disinhibition mechanism due to a reduced A-delta fiber input. Moreover, such an underlying mechanism could explain the distinctive pain exacerbation during or shortly after a period of physical stress.

### Limitations

To date, the pathogenicity of certain mutations in Fabry is discussed intensively. This discussion is currently ongoing; therefore, an evidence-based consensus statement of how to classify some of the “atypical” mutations is still lacking. In this study, all Fabry patients presented a mutation in the GLA-gene and concomitant extremity pain. However, although the majority of patients recruited (∼2/3) are classified with a pathogenic mutation, the outcome for few of the mutations included is still uncertain.

## Data Availability Statement

The datasets generated for this study are available on request to the corresponding author.

## Ethics Statement

The studies involving human participants were reviewed and approved by the Ethics Committee of the University Hospital of Kiel. Written informed consent to participate in this study was provided by the participants’ legal guardian/next of kin.

## Author Contributions

RB, JF, and PM conceived and designed the experiments. MS and MR carried out the experiments. JF, PH, and JGi performed the statistical analysis. RB and JF drafted the manuscript. JGa, SC-K, and SR provided critical suggestions to the study and final version.

## Conflict of Interest

JF reports grants from Sanofi Genzyme GmbH, during the conduct of the study; personal fees and non-financial support from Grünenthal GmbH and Sanofi Genzyme GmbH, personal fees from Bayer, non-financial support from Novartis, outside the submitted work. MS reports grants from Sanofi Genzyme GmbH, during the conduct of the study. PM reports grants from Sanofi Genzyme GmbH, during the conduct of the study; personal fees from Sanofi Genzyme GmbH, outside the submitted work. MR reports grants from Sanofi Genzyme GmbH, during the conduct of the study; personal fees from Pfizer, Grünenthal GmbH, and Astellas, grants from Mundipharma and Grünenthal GmbH, outside the submitted work. SC-K reports personal fees from Sanofi Genzyme GmbH, Shire, Amicus Therapeutics, and Chiesi, outside the submitted work. JGa reports personal fees from Sanofi Genzyme GmbH, Takeda, and Amicus, outside the submitted work. SR reports grants from Sanofi Genzyme GmbH, during the conduct of the study; personal fees from Bayer, outside the submitted work. PH reports grants from Sanofi Genzyme GmbH, during the conduct of the study; personal fees from Sanofi Genzyme GmbH, Pfizer, and Grünenthal GmbH, outside the submitted work. JGi reports grants from Sanofi Genzyme GmbH, during the conduct of the study; personal fees from Pfizer, Sanofi Pasteur, and Grünenthal GmbH and Glenmark Pharmaceuticals, outside the submitted work. RB reports personal fees from Pfizer Pharma GmbH, Genzyme GmbH, Grünenthal GmbH, Mundipharma Research GmbH und Co. KG, Allergan, Sanofi Pasteur, Medtronic, Eisai, Lilly GmbH, Boehringer Ingelheim Pharma GmbH & Co. KG, Astellas Pharma GmbH, Novartis Pharma GmbH, Bristol-Myers Squibb, Biogenidec, AstraZeneca GmbH, Merck, Abbvie, Bayer-Schering, MSD GmbH, Daiichi Sankyo, Glenmark Pharmaceuticals S.A., Seqirus Australia Pty. Ltd., Teva Pharmaceuticals Europe Niederlande, Teva GmbH, Genentech, Mundipharma International Ltd. United Kingdom, Astellas Pharma Ltd. United Kingdom, TAD Pharma GmbH, Galapagos NV, Kyowa Kirin GmbH, Vertex Pharmaceuticals Inc., Biotest AG, Celgene GmbH, Desitin Arzneimittel GmbH, Regeneron Pharmaceuticals Inc. United States, Theranexus DSV CEA Frankreich, Grünenthal SA Portugal, Abbott Products Operations AG Schweiz, Bayer AG, Grünenthal Pharma AG Schweiz, Mundipharma Research Ltd. United Kingdom, Akcea Therapeutics Germany GmbH, Asahi Kasei Pharma Corporation, AbbVie Deutschland GmbH & Co. KG, Air Liquide Sante International Frankreich, Alnylam Germany GmbH, Lateral Pharma Pty Ltd., Hexal AG, Ethos Srl Italien, Janssen, Sanofi-Aventis Deutschland GmbH, Agentur Brigitte Süss, Grünenthal B.V. Niederlande and grants/research support from EU Projects: “Europain” (115007). DOLORisk (633491). IMI Paincare (777500). German Federal Ministry of Education and Research (BMBF): Verbundprojekt: Frühdetektion von Schmerzchronifizierung (NoChro) (13GW0338C). German Research Network on Neuropathic Pain (01EM0903). Pfizer Pharma GmbH, Genzyme GmbH, Grünenthal GmbH, Mundipharma Research GmbH und Co. KG., Alnylam.
